# 2120: Antipsychotic-induced weight gain arises, in part, from alteration of feeding circuitry in the lateral hypothalamic area

**DOI:** 10.1017/cts.2017.22

**Published:** 2018-05-10

**Authors:** Ryan Michael Cassidy, Hannah Savage, Yungang Lu, Xiang Yang Zhang, Qingchun Tong

## Abstract

OBJECTIVES/SPECIFIC AIMS: To demonstrate that olanzapine recapitulates the effect of increased lateral hypothalamic (LH) GABAergic activity in the DRN and the DBB. This will provide a potential neural substrate for the observed increase in consumption of food and weight gain. METHODS/STUDY POPULATION: (1) We will examine electrophysiological activity of the DRN and the DBB in response to optogenetic stimulation of LH fibers to these nuclei. (2) We will identify the behavioral phenotype of stimulating these same projections using optogenetic techniques. (3a) Identify the behavioral phenotype of mice possessing cre-loxp-dependent knockout (KO) of LH GABAergic activity, DRN serotonergic activity, and inhibition of DBB cholinergic activity. (3b) Using these mice, we will establish behavioral response to olanzapine in ad libitum feeding and fast-refeeding condition. (4) Using baseline and post-treatment body mass index (BMI), PANSS, and side effect profile scores from a recently completed prospective cohort study of treatment-naive schizophrenic patients receiving atypical antipsychotics for 1 year, we will sequence multiple single nucleotide polymorphisms and explore the correlation of serotonergic, dopaminergic, and cholinergic receptor mutations with the increase in BMI and changes in PANSS score and side effect scores. RESULTS/ANTICIPATED RESULTS: (1) Our preliminary data indicates that the LH exclusively sends GABAergic input to the DBB, and the large majority of its projections to the DRN are GABAergic. (2) We have identified that stimulating LH–>DBB projections produces intense feeding and drinking behavior, a real-time place preference for laser stimulation, and a conditioned place preference for laser stimulation. Preliminary data shows that the LH->DRN also produces feeding behavior. (3a) Our lab has demonstrated that transgenic mice with LH-specific GABA release KO are smaller, have increased anxiety-like behaviors such a repetitive grooming and open field aversion, and have reduced feeding after fasting conditions. We expect the DRN serotonergic KO mice to have increased body weight and reduced anxiety-like behaviors. (3b) Our pilot study demonstrated that the LH GABA KO mice administered olanzapine have a greater consumption of food over 1 hour than controls (n=7, 5, respectively; *p*=0.08). DRN serotonergic KO mice and mice with inhibition of choline will have an increased baseline feeding behavior, but will not be affected by olanzapine. (4) We believe that SNPs in serotonergic receptors such as 5HT2C, and those affecting dopaminergic and cholinergic receptors, will be more common in schizophrenic patients with increased BMI than those without. Further, we believe that a reduction in the PANSS items reflecting anxiety and aversiveness will correlate with increased BMI, since we postulate that mimicking LH GABAergic activity will produce its previously demonstrated anxiolytic effects. DISCUSSION/SIGNIFICANCE OF IMPACT: Identifying the important role for a reward-oriented feeding center in the brain in producing antipsychotic weight gain will allow a more comprehensive, ethologically sound approach to behavioral modification therapy in these patients. It will lend mechanistic credence to weight control therapies which have used token economy, opioid antagonism, and other inhibition-promoting therapies. This study will also increase the validity for testing further the use of selective serotonin agonists which prevent weight gain such as lorcaserin.

